# Meaningful Memory? Eighteen-Month-Olds Only Remember Cartoons With a Meaningful Storyline

**DOI:** 10.3389/fpsyg.2018.02388

**Published:** 2018-11-28

**Authors:** Trine Sonne, Osman S. Kingo, Peter Krøjgaard

**Affiliations:** Department of Psychology and Behavioral Sciences, Center on Autobiographical Memory Research, Aarhus University, Aarhus, Denmark

**Keywords:** infant memory, storyline, eye-tracking, visual paired-comparison, meaningfulness, dynamic material

## Abstract

In two studies we investigated the importance of a storyline for remembering cartoons across a delay of 2 weeks in 18-month-old infants by means of the visual paired-comparison (VPC) paradigm. In Study 1 seventy-one 18-month-olds were tested using similar cartoons as in a recent study from our lab while varying the richness of the storyline information. In a VPC task half of the infants watched uncompromised versions of the cartoons used in the recent study (Storyline Condition), whereas the other half watched Pixelized versions of the cartoons (number of pixels reduced by 98% covering up the narrative, but leaving perceptual details, e.g., colors, movements, the same, and Pixelized Condition). Two weeks later they were presented with the familiar cartoon and a novel cartoon from the same version (Storyline or Pixelized) simultaneously, while being eye-tracked. Results showed that only the infants in the Storyline Condition remembered the target cartoon, thus suggesting that the storyline is important for memory. However, an alternative interpretation of the results could be that what made the infants in the Storyline Condition remember the target cartoon was not the storyline, but the static conceptual information of the objects and agents present in the cartoon (which was not visible in the Pixelized version). To test this possibility, a control study was created. In Study 2 thirty-six infants were therefore presented with a version of the cartoon in which we broke down the temporal presentation into 1 s segments and presented these out of order. This was done to preserve the static conceptual information (e.g., objects and agents) while still disturbing the storyline. Results showed that the infants in this condition still did not remember the target cartoon, suggesting that the meaningfulness of the storyline – and not only static conceptual information – is important for later memory.

## Introduction

The visual paired-comparison (VPC) paradigm has been used extensively over the years to document that infants are indeed capable of remembering visual material over shorter or longer delays (e.g., from a few minutes to several weeks, see e.g., Fantz, 1964; Fagan, 1974; Kingo and Krøjgaard, 2015; Hayne et al., 2016; Sonne et al., 2016a). The VPC paradigm has been used in different formats, but the typical procedure has been to present infants with visual material for a specific time period (a familiarization phase), and then after a delay to test their memory of this material by presenting the infants with the now familiarized material next to a novel stimulus (see e.g., Hayne, 2004).

Memory has typically been inferred when the infants look longer at the novel stimulus compared to the familiar one. This interpretation is following Sokolov (1963) comparator theory of the orienting response describing that we orient our attention to stimuli until an internal model has been made. Once this model is complete, our attention will shift toward new stimuli indicating that the familiarized stimulus is still in our memory. However, following the more recent model put forward by Bahrick and Pickens (1995), both novelty and familiarity preferences should be taken as sign of recent and remote memory, respectively, and null preferences should not necessarily be interpreted as forgetting. Today, several studies have shown that familiarity preferences can be taken as valid evidence of memory as well (e.g., Kingo and Krøjgaard, 2015; Hayne et al., 2016; Sonne et al., 2016a, 2017).

Although the VPC paradigm has been used widely to document memory in infancy, we still do not understand exactly *which aspects* of the stimulus material that are memorable for infants. When testing infant memory using the VPC paradigm, the majority of the studies have used static material such as photographs (e.g., of a chair or a face, e.g., Fagan, 1974; Bushnell, 2001; Hayne et al., 2016), or abstract patterns (e.g., Fantz, 1964; Fagan, 1974). For methodological reasons, the use of simple, static stimuli was an obvious place to start testing visual recognition memory using the VPC. However, the infant world contains much more than static stimuli of (unfamiliar) objects or patterns. Infants are constantly exposed to dynamic scenes that unfold over time of which they have to make sense and at times remember. Taking such considerations into account, researchers have more recently looked into the nature of the stimuli by directly testing infants’ memory of material with a special meaning to them (e.g., a photo of the infant’s own mother, house, car, and security object, see Hayne et al., 2016) or by using dynamic material (i.e., stimuli involving movements) when employing the paradigm (e.g., Bahrick and Pickens, 1995; Richmond et al., 2007; Morgan and Hayne, 2011; Kingo et al., 2014).

The massive amount of studies conducted employing the VPC paradigm are thus characterized by substantial variations in the complexity as well as in the meaningfulness of the stimuli used, varying from simple still pictures to dynamic event sequences involving intentionality, actions, and goal-directedness. If we are to understand when and how infants remember events from everyday life, it seems crucial that researchers move on to use meaningful and dynamic material when assessing memory using the VPC paradigm, because such material is more similar to the events infants experience in their everyday lives, and in addition motion is providing important information of events (see also Bahrick and Pickens, 1995). Inspired by this line of argument, a recent study from our lab showed that 18-month-old infants, in a VPC task, were capable of remembering complex dynamic material (i.e., a cartoon involving agents and a plot or a storyline) across a delay of 2 weeks (Kingo and Krøjgaard, 2015). This was evidenced by more looking time directed at the familiar cartoon. Importantly, the results from this study moreover indicated that the infants’ understanding of the storyline was important for their later memory of this material. Based on these results it was suggested that the infants’ ability to understand the storyline in the cartoon was important for later memory (Kingo and Krøjgaard, 2015). This was, however, only inferred and to our knowledge the importance of a storyline for later memory has not yet been systematically investigated. By the term *storyline*, we refer to an event involving agents and objects that unfolds in time and displays a simple sequence of actions containing a beginning and an endpoint. A storyline therefore could involve a causal sequence of events, but we do not consider causally ordered sequences to be a necessary component for a sequence to display a storyline.

Although only little is known regarding infants’ comprehension of movie sequences (Pempek et al., 2010), there is evidence to suggest that infants comprehend simple movie sequences. For instance, Pempek et al. (2010) presented 6-, 12-, 18-, and 24-months-old infants with normal and distorted excerpts from the TV program *Teletubbies*, while assessing their looking time and heart rate. In the normal version, the movie was used as is. In the distorted versions, two kinds of distortions were employed: in one version, the speech track was reversed for each utterance (making the speech incomprehensible); in the other version, the temporal order of the movie was manipulated by re-arranging movie segments of app. 5 s duration throughout the movies. The results revealed that whereas 6-, and 12-month-olds devoted the same amount of looking time toward the normal and distorted movie segments, the 18- and 24-month-olds looked reliably longer at the normal movie sequences relative to the distorted versions. These results suggest, that around 18 months of age, infants begin to comprehend simple movie sequences.

Converging evidence had also been obtained in an earlier study by Richards and Cronise (2000) in which infants in the 6- to 24-months age range were exposed to (a) a sequence from the TV program *Sesame Street*, and (b) visual computer-generated abstract patterns mixed with elements from the *Sesame Street* program. The amount of visual fixations were assessed for both stimuli. Again, the results revealed that only infants 18 months of age or above looked reliably longer at the *Sesame Street* segments, relative to the computer-generated abstract patterns. Taken together these results suggest that around 18 months of age, infants seem to comprehend simple movie material (see also Baldwin et al., 2001; Anderson and Hanson, 2010 for an example of even younger infants’ demonstration of a sensitivity to the inherent structure in intentional actions performed by actors in movies). However, we do not know whether storyline information also facilitates infants’ ability to *remember* such movie sequences over time. In the present study we set out to explore this question.

## The Present Study

In order to investigate the importance of a storyline when using complex dynamic material, we here, by means of the VPC paradigm, present two studies examining infant memory of cartoons while varying the richness of the storyline information. In Study 1 18-month-old infants were randomly allocated to one of two conditions: The Storyline Condition or the Pixelized Condition. In the Storyline Condition (storyline maintained) we set out to replicate the results from previous studies from our lab in which children in the age range from 16- to 20 months of age at test displayed a clear familiarity preference for the cartoon encountered during encoding, using the same stimulus material and the same design (with a few minor modifications) (Kingo and Krøjgaard, 2015; Sonne et al., 2016a). In the Pixelized Condition (storyline disrupted), we examined whether the infants would remember the cartoon when presented with a version of the same cartoon in which the storyline information was severely attenuated. This was done by reducing the number of pixels by 98%, resulting in a cartoon in which the duration, movements, and colors were preserved, but where the storyline had disappeared because of the pixelization. If – as indicated by the previous study from our lab – the storyline *is* important for later memory, then our expectations would be that these infants would not show sign of memory. If, however, the storyline is not important as such, then the infants could very well show sign of remembering this cartoon for instance due to memory of colors or movements in the cartoon (see for instance Richards, 1997; Courage and Howe, 1998 for studies illustrating memory in infancy for patterns or abstract figures).

## Study 1

### Methods

#### Participants

Seventy-one 18-month-olds (*M*_age_ = 18.08 months, *SD* = 0.21 months, range 17.6–18.6 months, 31 girls) participated in the first study. The infants were recruited from birth registries from the National Board of Health and were predominantly Scandinavian Caucasian from the area of Aarhus living in families with middle to a higher SES. All of the infants were healthy and had an Apgar score ≥7. Informed and written parental consent was obtained at the first visit. An additional 23 infants were tested, but later excluded: three due to fussiness, two due to a technical error, one due to an experimenter error, two due to parental interference, and 15 due to limited looking at the cartoons, either because they did not pay attention to at least one presentation during encoding (assessed by the experimenter) or their looking time (at test) being 2 SDs below the overall group mean. The study was approved by the local Ethics Committee at Center on Autobiographical Memory Research, Department of Psychology and Behavioral Sciences.

**FIGURE 1:**
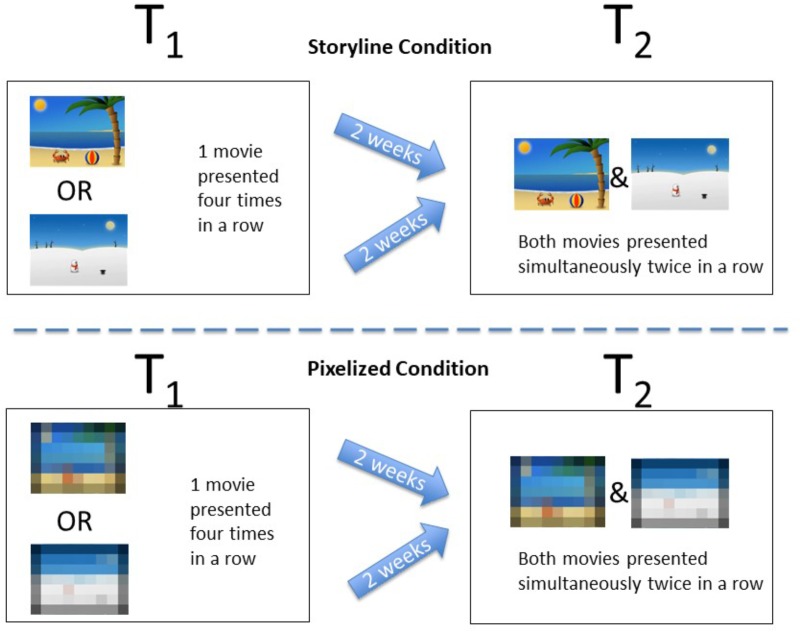
Schematic depiction of the design and procedure from Study 1 including static pictures from the cartoons used in the two conditions.

The test group was randomly divided into two conditions: (1) the Storyline Condition (*N* = 38), in which they were presented with the exact identical cartoons used in the previous study from our lab, (2) the Pixelized Condition (*N* = 33) in which they were presented with a cartoon with the number of pixels reduced by 98% (see Figure 1).

#### Materials

**FIGURE 2:**
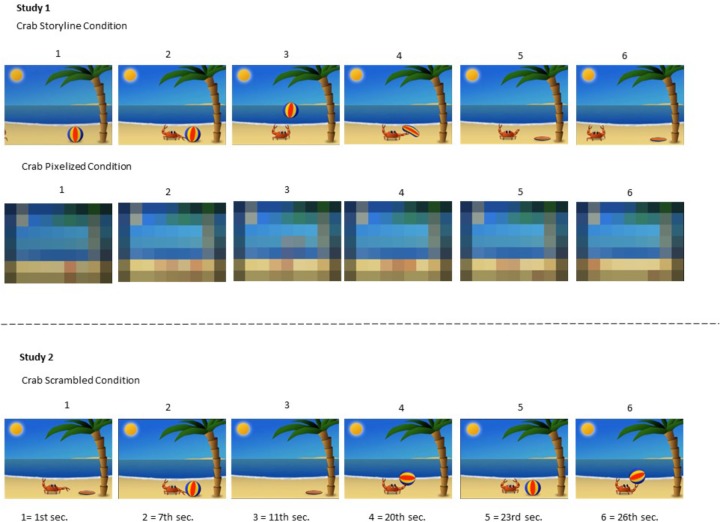
Static pictures (Crab Versions only) illustrating the time flow of the cartoons from both studies.

Two animated and custom-made cartoons of 30 s duration were used for this study. Almost identical versions of the cartoons have been used in previous studies from our lab (Kingo and Krøjgaard, 2015; Sonne et al., 2016a, 2017). One cartoon was about a crab and the other about a snowman. In the crab cartoon the crab entered a beach-like scene from the left side, then started playing with a ball until one of the claws punctured the ball causing it to deflate. Finally, the crab exited the scene toward the right side. In the snowman cartoon the snowman entered a winter landscape from the left side, then started jumping up and down making a hat bounce until it landed on top of the snowman’s head. Finally, the snowman exited toward the right side. For the Pixelized versions of the cartoons the number of pixels was simply reduced by 98%. This was done to prevent the infants from being able to see the agents, objects, actions and scenarios, while preserving the same length, luminance, color, and movement as in the Storyline version of the cartoon (see Figures 1, 2). All cartoons were made without an audio track to avoid overlapping sounds when presenting two cartoons simultaneously at test.

To validate whether our manipulation worked, that is, whether we succeeded in reducing the richness of the storyline information in the Pixelized version, a group of adults (*N* = 33) were asked whether they could identify what was happening in these cartoons. Although very few of them guessed that the crab cartoon was taking place at a beach, more importantly no one guessed what the cartoons were about, or were capable of identifying the agents in the cartoons.

In addition, the parents were asked to fill out the Danish version of the MacArthur-Bates Communicative Development Inventory (CDI).

#### Design and Procedure

At the first visit all of the infants were familiarized to one 30 s cartoon (snowman or crab, either in the Storyline Condition or in the Pixelized Condition). Following the procedure used in previous studies using similar stimuli (Kingo and Krøjgaard, 2015; Sonne et al., 2016a), the cartoon was presented four times in a row (120 s) to ensure encoding. After a two-week delay (*M* = 13.94 days, *SD* = 0.86 days) they returned to our lab, and following the VPC paradigm they were presented with the now familiar cartoon next to a novel cartoon (from the same cartoon category) two times in a row while their eye movements were tracked (see Figure 1). The left/right presentation of the cartoons was counterbalanced. Based on the results from the previous studies from our lab, we expected that the infants in the Storyline Condition would show sign of remembering the target cartoon evidenced by a familiarity preference (see e.g., Kingo and Krøjgaard, 2015; Sonne et al., 2016a). If the storyline is important for infant memory, then we would expect no sign of memory for the infants in the Pixelized Condition. If, however, other factors that were preserved across the alteration of the cartoons such as colors, movement details, or luminance would be enough to create lasting memory of the cartoon, then we would expect the infants in the Pixelized Condition to show sign of memory as well.

#### Eye-Tracking Setup and Data Extraction

To register the infants’ visual preferences during the test, we used a Tobii X120 (Tobii Technology, Stockholm) that recorded fixations at 120 Hz with 0.5° accuracy on a 30″ LCD monitor. The total visual angle of the screen was 40° (width) × 25° (height), whereas the visual angle of the stimuli area was 33° (width) × 16.5° (height). The distance between the eye tracker and the eyes of the infant was approximately 70 cm. For the data collection we used the Tobii Fixation Filter (default). Initially, a five-point calibration procedure was conducted using the Tobii Studio calibration for infants. The cartoons were presented by use of E-prime software (Psychology Software Tools, United States).

Provided that our interest was to know more about the infants’ general looking time at the two cartoons, respectively, two simple areas of interest were created covering each of the cartoons. Within these areas of interest we assessed fixation duration providing us a measure of the infants’ absolute looking time within these defined areas.

### Results and Discussion

To ensure that the infants in the two conditions were equivalent, we initially investigated whether there were any differences in relation to their productive language skills. A *t*-test revealed that there were no overall differences in productive language between the two conditions: *t*(69) = 0.34, *p* = 0.74, *r* = 0.04 (*M*_Storyline_ = 73.47 words, *SD* = 65.09 words; *M*_Pixelized_ = 68.36 words, *SD* = 60.78 words).

To investigate whether the infants remembered the cartoon they had been familiarized to previously, we used the standard way of analyzing this when employing the VPC paradigm (e.g., Roder et al., 2000; Richmond et al., 2007). We calculated the proportional looking to the novel cartoon by dividing absolute looking time to the novel cartoon with total looking time at both cartoons, thereby leaving us with a number between 0 and 1.

Preliminary analyses revealed no gender differences in relation to absolute looking time during test or proportional looking to the novel cartoon when looking at the two conditions, and the data was therefore collapsed over gender in the following analyses. We, however, saw a significant difference in relation to absolute looking time during test in the two different conditions, reflecting more looking time in the Storyline Condition compared to the Pixelized Condition: *t*(69) = 9.27, *p* < 0.001, *r* = 0.74 (*M*_absolute_looking_Storyline_ = 45.71 s, *SD* = 10.09 s; *M*_absolute_looking_Pixelized_ = 21.41 s, *SD* = 12.00 s). This was to be expected when thinking about the nature of the stimuli (e.g., Anderson et al., 1981), and since (a) the infants were excluded if their looking time at test was 2 SDs below the overall group mean, and (b) the design was counterbalanced *within* each condition (see Figure 1), and (c) our main analyses focused on proportional looking, condition specific differences in encoding measures is no threat to the validity of the VPC design. We found no differences in absolute looking time at the two different cartoons in each condition: Storyline Condition: *t*(36) = 0.057, *p* = 0.955, *r* = 0.10 (*M*_absolute_looking_Snowman_ = 45.80 s, *SD* = 11.56 s; *M*_absolute_looking_Crab_ = 45.61 s, *SD* = 8.49 s), Pixelized Condition: *t*(31) = 0.190, *p* = 0.850, *r* = 0.034 (*M*_absolute_looking_Snowman_ = 21.88 s, *SD* = 11.99 s; *M*_absolute_looking_Crab_ = 21.07 s, *SD* = 12.33 s).

First, we wanted to test whether the infants in the Storyline condition indeed remembered the cartoon they had been familiarized to at the first visit. When comparing the proportional looking to the novel cartoon to chance level (0.5) we saw a clear familiarity preference, both when considering the two iterations combined (60 s): *t*(37) = −5.39, *p* < 0.001, *r* = 0.66 (*M* = 0.35, *SD* = 0.17), but also when looking at the two individual iterations of the cartoons during test: 1st iteration (30 s): *t*(37) = −5.16, *p* < 0.001, *r* = 0.65 (*M* = 0.34, *SD* = 0.19), 2nd iteration (30 s): *t*(37) = −4.396, *p* < 0.001, *r* = 0.59 (*M* = 0.35, *SD* = 0.21). The infants in the Storyline Condition thus showed clear evidence of recognizing the familiar cartoon, replicating previous findings from our lab (Kingo and Krøjgaard, 2015). The fact that we see a familiarity preference is also in accordance with our expectations based on previous studies from our lab using versions of the same cartoons (Kingo and Krøjgaard, 2015; Sonne et al., 2016a).

**FIGURE 3:**
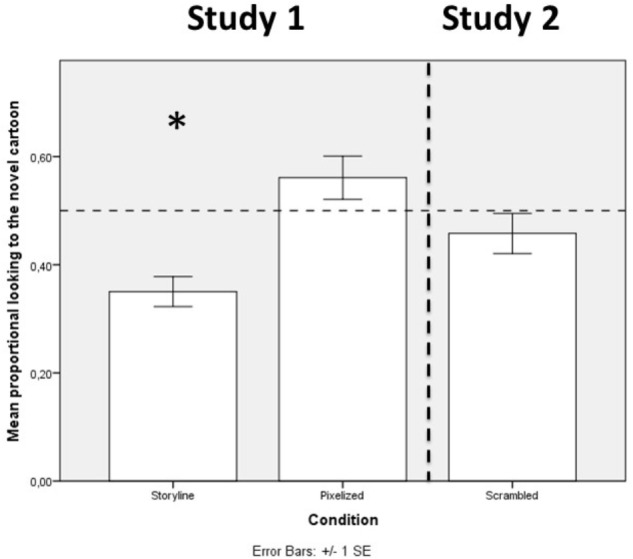
A graphical representation of the mean proportional looking to the novel cartoon for all three conditions from the two studies. The dotted line represents chance level at 0.5. An ^∗^ marks a significant difference at the 0.05 level.

Next, we wanted to investigate the importance of the storyline for infant memory. We therefore compared the proportional looking to the novel cartoon to chance level (0.5) for the infants in the Pixelized Condition. None of these analyses resulted in a significant difference from chance level [both iterations combined: *t*(32) = 1.53, *p* = 0.14, *r* = 0.26 (*M* = 0.56, *SD* = 0.23), 1st iteration: *t*(32) = 1.53, *p* = 0.14, *r* = 0.26 (*M* = 0.56, *SD* = 0.23), and 2nd iteration: *t*(32) = 1.55, *p* = 0.13, *r* = 0.26 (*M* = 0.58, *SD* = 0.29)]. There was thus no indication that the infants in the Pixelized Condition remembered the target cartoon across the 2 weeks (see Figure 3).

However, an alternative interpretation might be that the infants simply remembered the cartoons in the Storyline Condition, not due to the storyline *per se*, but due to the static conceptual information (e.g., scenarios, agents, or objects). To disentangle the two interpretations, a control study was created.

## Study 2

This study was designed as a control for the results from Study 1. One might argue that the Storyline Condition and the Pixelized Condition not only differed with regard to storyline, but also with regard to whether the conceptual content, such as actions, objects, and agents, was visible. In Study 2, in the same overall VPC task, we therefore set out to examine whether 18-month-old infants would remember the cartoon, if they were presented with a version of the cartoon in which the conceptual content was preserved, while the storyline (the temporal dynamics) was disrupted.

To do so, we broke down the temporal presentation of the cartoon and presented the infants with a *scrambled* order of the bits from the cartoon. This was done in order to disrupt the storyline, while preserving the static conceptual information such as agents, scenarios, and objects. We had no firm hypotheses regarding this study; however, if the storyline *is* important for later memory, this group of infants may not show sign of remembering the cartoons at all. If information of agents, objects, etc., is sufficient to make the infants remember, then they may show a familiarity preference.

### Methods

#### Participants

In this Study thirty-six 18-month-olds (*M*_age_ = 18.27 months, *SD* = 0.33 months, range 17.7–18.7 months, 21 girls) participated. The infants were recruited from the same registries using the same criteria and were also from the area of Aarhus. Again informed and written parental consent was obtained. Six additional infants were tested but later excluded: 1 due to looking time (at test) being 2 SD below the overall group mean, 2 due to fussiness, and 3 due to technical errors.

#### Materials

In Study 2 the exact same cartoons as described in Study 1 for the Storyline Condition were used. However, this time we broke down the temporal presentation of the cartoons by dividing them into smaller segments with a duration of 1 s each. Then we arbitrarily (while ensuring that no adjacent segments maintained their original order) joined the segments into new cartoons in which the storyline now was disrupted, while preserving the static conceptual information of agents, objects, and scenarios. The children were therefore presented with versions of the cartoons in which the order of the actions and activities were shuffled, hence disturbing the inherent storyline of the cartoon. This was referred to as the Scrambled version of the cartoons. The parents were again asked to fill out the Danish version of the MacArthur-Bates CDI.

#### Design, Procedure, and Eye-Tracking

The procedure was identical to that described in Study 1 except in this study half of the infants were presented with the Scrambled version of the snowman cartoon and the other half were presented with the Scrambled version of the crab cartoon. The infants were thus again familiarized to one of the cartoons and after 2 weeks (*M* = 13.78 days, *SD* = 1.12 days) they returned for the memory test. Eye tracking set-up and data extraction were also done in the same way as described above.

### Results and Discussion

A one-way ANOVA revealed no difference in relation to vocabulary scores when comparing the infants from this condition to the infants from the two conditions in Study 1: *F*(2, 103) = 0.229, *p* = 0.796^1^, partial Eta-Squared = 0.004 (Exp. 1: *M*_Storyline_ = 73.47 words, *SD* = 65.09 words; *M*_Pixelized_ = 68.36 words, *SD* = 60.78 words, Exp. 2: *M*_Scrambled_ = 63.14 words, *SD* = 69.23 words). Therefore we considered the infants from this Study to be comparable to the infants from Study 1.

Again preliminary analyses revealed no gender differences in relation to absolute looking time or proportional looking to the novel cartoon and the results were therefore collapsed over gender for the following analyses. Additionally, no differences were found in relation to absolute looking time at the two different cartoons in this condition: *t*(34) = −0.09, *p* = 0.93, *r* = 0.015 (*M*_absolute_looking_Snowman_ = 46.91 s, *SD* = 8.996 s; *M*_absolute_looking_Crab_ = 47.19 s, *SD* = 9.87 s).

Repeating the analytic strategy from Study 1, we compared the proportional looking to the novel cartoon to chance level (0.5) for the infants in the Scrambled Condition. None of these analyses resulted in a significant difference from chance: [proportional looking at both iterations (*M* = 0.46, *SD* = 0.22) compared to chance level (0.5): *t*(35) = −1.13, *p* = 0.27, *r* = 0.19 proportional looking at the 1st iteration (*M* = 0.44, *SD* = 0.24) compared to chance (0.5): *t*(35) = −1.53, *p* = 0.14, *r* = 0.25, and finally proportional looking at the 2nd iteration (*M* = 0.47, *SD* = 0.28) compared to chance level (0.5): *t*(35) = −0.57, *p* = 0.57, *r* = 0.26]. There was thus no indication that the infants in the Scrambled Condition remembered the cartoons across the 2 weeks (see Figure 3).

These results suggest that information concerning static conceptual features is not sufficient for the infants to remember the target cartoon in this study. However, to rule out other explanations as to why the infants watching the Pixelized and the Scrambled versions of the cartoons did not remember them after 2 weeks, we ran some additional analyses.

#### Additional Analyses

Recall, that precautions had been made to ensure that the infants attended to the material during both encoding and test (i.e., only infants who attended to at least one of the four presentations during encoding (as assessed by the experimenter) were included, and only infants for whom their looking time at test was within 2 SDs of the overall group mean were included). However, strictly speaking, the infants’ looking time was only quantified during test, but not during encoding, and one might speculate whether the failure to demonstrate memory at test for the infants in the Pixelized Condition and in the Scrambled Condition may not have been caused by forgetting as such, but simply were due to insufficient attention during encoding (i.e., only encoded material can subsequently be remembered). As described in the introduction we know that distorted versions of TV shows for kids have an effect on infants’ looking time during encoding (Anderson et al., 1981; Pempek et al., 2010), but we do not know whether this affects later memory. Other studies, however, have shown that looking time during the encoding of an event is not necessarily a strong predictor to later memory (e.g., Sonne et al., 2016b). We therefore wanted to investigate whether our results could be explained by differences in looking time during encoding. Although this was not part of the original focus of the Study we decided to include this as additional analyses.

**FIGURE 4:**
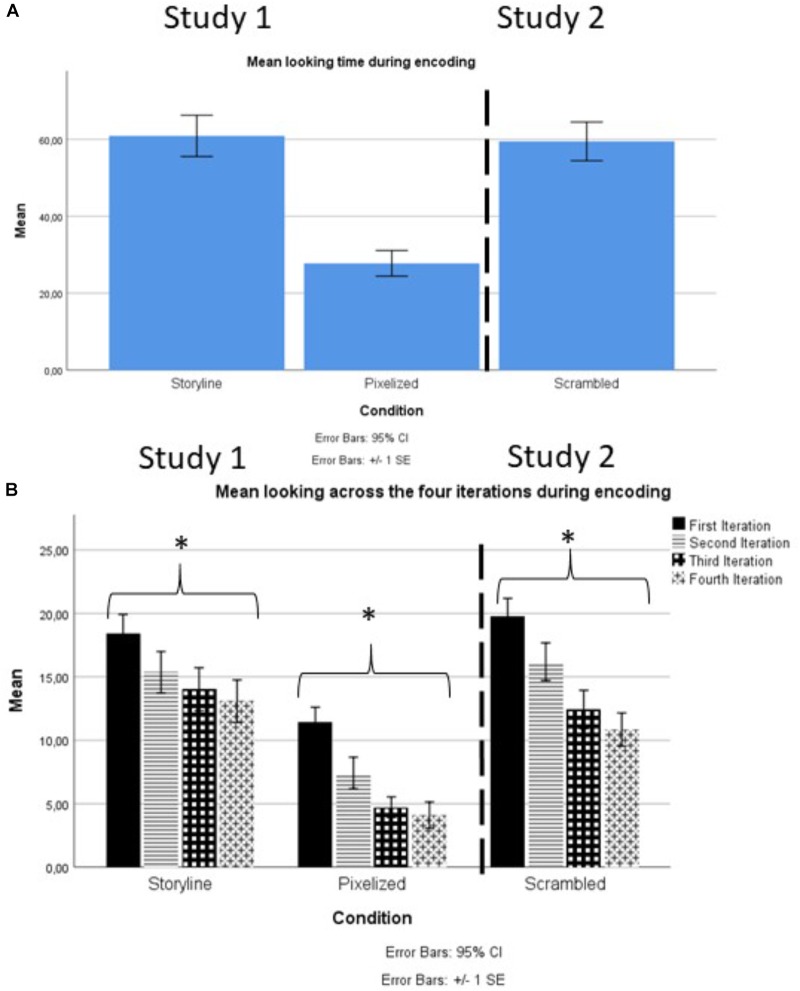
**(A,B)** A graphical representation of the mean looking time during encoding depicted as **(A)** total looking time during encoding and **(B)** looking time from each of the four repeated presentations of the target cartoon during encoding. An ^∗^ marks a significant difference at the 0.05 level.

A graphical representation of the encoding data is displayed in Figure 4 for visual inspection^2^.

Looking at Figure 4A we see that although the infants in the Pixelized Condition looked less at the material than the other two groups (as was to be expected based on previous studies on attention, e.g., Pempek et al., 2010), the looking time during encoding in the Scrambled Condition is actually very similar to the looking time in the Storyline Condition. This means that increased devoted attention during encoding does not ensure subsequent memory. Moreover, we also see a clear indication that the children encoded the material presented to them, as they all – *regardless* of condition – habituated to the material as evidenced by the systematic, gradual decline in looking time across the four different iterations of the target cartoon (see Figure 4B). Repeated measures ANOVA with looking time across the four iterations of the cartoons as a within-subjects factor for each condition separately on absolute looking time, confirmed this impression. For all three conditions, the decline in looking time across the four encoding trials was clearly significant (Storyline Condition: *F*[3, 102] = 4.66, *p* = 0.004, partial Eta-Squared = 0.121; Pixelized Condition: *F*[3, 72] = 19.04, *p* < 0.001, partial Eta-Squared = 0.442; Scrambled Condition: *F*[3, 102] = 25.74, *p* < 0.001, partial Eta-Squared = 0.431). The encoding data thus provide evidence that the infants did indeed encode the trials in all three conditions during the first visit. Consequently, the obtained differences in the results at test 2 weeks later cannot be explained by insufficient encoding, but have to be attributed to differences in memory.

## General Discussion

Taken together, the results from the present two studies showed that the infants’ memory was dependent on the meaningfulness of the storyline, as only the infants who were presented with the versions that – from an adult point of view – had a coherent and meaningful storyline showed sign of remembering the target cartoon. Additional analyses also showed that the lack of memory for the infants in the two conditions with altered storylines could not be explained by a lack of attention during encoding, as all of the infants – no matter what condition they were in – habituated systematically to the material.

The finding that a storyline is of importance for 18 month-olds’ memory of event sequences is in accordance with a whole line of studies suggesting that infants quite early are capable of understanding events or activities much along the same way as adults do (e.g., Baldwin et al., 2001; Saylor et al., 2007; Pace et al., 2013). Baldwin et al. (2001) for instance showed how even 10–11 month-olds process dynamic action much in line with adult processing. By presenting infants with videos of everyday events they documented that infants after being familiarized to the material subsequently would look longer at the videos if the actors were paused while performing an activity, but not if they were paused after such activity had been carried out. This was taken as evidence that infants parse dynamic action according to a sensitivity to the structure of intentional actions. These results are supported by the findings from a line of literature suggesting that even infants have a basic understanding of intentional aspects of human behavior (e.g., Woodward et al., 2001).

Moreover, recent evidence suggests that infants not only understand and parse event sequences much like adults, but that this ability also has consequences for their *memories* of these events (e.g., Sonne et al., 2016a, 2017) – following what has been found for adults (e.g., Swallow et al., 2009). Sonne et al. (2016a) for instance investigated whether 16- and 20-month-olds would react to disturbances placed in a cartoon. The disturbances were created by having ellipses covering information from the cartoon for 3 s out of the total 30 s cartoon. Following *Event Segmentation Theory* (see e.g., Kurby and Zacks, 2008; Swallow et al., 2009) – a theory suggesting that especially information at event boundaries (or break points) between event units is important for adult memory – it was predicted that the disturbances would have different consequences for the infants’ memory of the cartoon depending on their temporal placement. The results suggested that indeed even in infancy, boundary information is important for memory, as evidenced by the finding that the infants who watched a cartoon with ellipses inserted at event boundaries showed a reduced familiarity preference compared to the infants with ellipses inserted at other time points. Interestingly, this pattern of result was not as pronounced for the 16 month-olds as for their 20-month-old peers (Sonne et al., 2016a).

A shared feature of the developmental studies just cited is that they all attempted to shed light on infants’ understanding of dynamic events unfolding over time. The importance of taking a departure point in stimulus material that resembles the everyday lives of infants has even shown its merits within infants’ perception – a field of research in which stimuli for methodological reasons are typically meticulously detached from their natural environment and examined in isolation. For instance, Bahrick et al. (2002) showed that three-month-old infants were better at detecting changes in tempo of rhythms when having bimodal access to the stimuli as opposed to unimodal access alone. Although unimodal paradigms are necessary with regard to control when investigating perception, the results from Bahrick et al. (2002) clearly demonstrate the importance of keeping in mind that unimodal access to stimuli is probably the exception in real life situations, and that infant research based on very simple and isolated stimuli may be at risk of underestimating the competencies of infants. Similarly, although in a slightly different domain, infants’ face recognition has been shown to improve when faces are presented dynamically instead of just statically (for a recent review, see Xiao et al., 2014).

Besides contributing to our understanding of infants’ conception of storylines, the findings from the present study may also have methodological implications. The finding that when using complex dynamic material the infants had a familiarity preference adds further evidence to the studies suggesting that not only novelty preferences should be taken as indication of memory. The evidence seems to suggest, that the preferences the infants show when using the VPC paradigm may be tied to the type of material (e.g., complex dynamic material) used during encoding (for similar arguments see Hunter and Ames, 1988) and to the experience of meaningfulness connected to the material (e.g., Hayne et al., 2016). Therefore, the evidence presented here may be of importance for future interpretations of the preferences documented when using the VPC paradigm.

Several questions deserve attention in future research. First, how early in the ontogenesis are infants capable of remembering cartoons as used in the present study or similar cartoons including a storyline? While several studies (Kingo and Krøjgaard, 2015; Sonne et al., 2016a) including the present one have shown that these specific cartoons containing storyline information can indeed be remembered by young infants (down to at least 16-month-olds, Sonne et al., 2016a), we do not know if younger infants would be capable of remembering them as well. Second, and along the same path, would memory of these cartoons be preserved across longer retentions intervals? As all previous studies have used no longer than a two-week retention interval, this is unknown for the time being. However, with three studies showing strong and stable evidence of memory of the cartoons involved, this stimulus material seem highly suitable for manipulating the retention interval. Third, it would be interesting to investigate whether these results would replicate using different controls, e.g., adding another control to the Scrambled version, that would still provide information regarding agents, actions, etc., but that did not involve abrupt visual changes caused by the scrambling of the cartoons. Although, we consider it unlikely that abrupt visual changes (which is difficult to avoid when scrambling movie sequences) by themselves should prevent infants from remembering the sequences, we cannot rule out this possibility when considering the existing evidence.

Fourth, and in a broader perspective: what are the critical features of the storylines that facilitate memory? Is it the story component *per se*, that is, the coherent sequencing of mini-events that unfolds over time? And are agents necessary ingredients in such storylines? In principle, an ordered and coherent sequence of events could take place exclusively in the inanimate domain (e.g., a ball rolling turning over pins). For instance, even 6-month-old infants appear to understand simple causal-effect relationships (i.e., when a box moves into another box, it may cause the second box to move; Leslie and Keeble, 1987), and elicited imitation studies with 20-month-olds have shown that action sequences are easier to remember when the order of the steps involved are physically constrained (so-called ‘enabling relations,’ e.g., Bauer, 1992). We cannot know whether the cartoons used in the Storyline Condition were easy to remember because of (a) their ordered sequences (i.e., in the crab cartoon, it is only possible to play with the ball *as* a ball, *before* it is punctured, not after), (b) because active and intentional agents (i.e., the crab and the snowman) were conducting meaningful and intended acts, or (c) a combination of these factors. In order to disentangle these possibilities, further studies are needed. It might be argued, however, that the stimulus material used in the present studies was somewhat artificial, simply because the material used was cartoons and not movies of the real world. This is true, of course, and it could be interesting indeed to examine whether the results would replicate if real movies with equivalent content involving human beings were used. On the other hand, other studies have used cartoons as media for examining the understanding of human agency with success in both infants (e.g., Johnson et al., 2007; Scola et al., 2015) and children (e.g., Abell et al., 2000). And even if cartoon material is actually less facilitating for memory than movies from the real world, it only suggests that infants may be even more sensitive to storyline information than the evidence from the present study suggests.

Taken together, the findings from this study suggest that a storyline is of importance when infants are to remember dynamic material across a delay of two-weeks. The infants seemingly quickly detected aspects of the cartoons related to agency and intentionality, and more importantly, this had consequences for their ability to remember such complex material. As such, the present study adds to the line of studies examining infant memory and cognition using material that is more in accordance with what they experience in their everyday infant lives than the majority of earlier research.

## Ethics Statement

This study was carried out in accordance with the recommendations of the local ethics committee at Center on Autobiographical Memory Research with written informed consent from all subjects. All subjects gave written informed consent in accordance with the Declaration of Helsinki. The protocol was approved by the local ethics committee.

## Author Contributions

All authors were responsible for the overall design and idea behind the present study. TS supervised the testing of the participants which was carried out by student assistants. TS and OK carried out the statistical analyses. TS was responsible for the final write-up of the paper. Preliminary drafts of the manuscript were commented on critically by all authors until agreement was reached.

## Conflict of Interest Statement

The authors declare that the research was conducted in the absence of any commercial or financial relationships that could be construed as a potential conflict of interest.

## Footnotes

1 Please notice that we failed to obtain CDI-scores from one participant.

2 Note that although these data had been collected during encoding, they were not initially extracted due to our main focus on subsequent memory. Here, we take them in consideration in order to rule out alternative interpretations of the results obtained at test. Note further, that because the data from the encoding sessions were not considered part of the study originally, the infants were not excluded if they for some reason did not provide data for this first visit (e.g., due to difficulties calibrating, eye-tracking fall-outs, and technical challenges). Therefore, in the provided additional analyses, the number of participants is a bit lower than when looking at data from the test session.
